# The importance of controlled clinical trials with extracellular vesicles

**DOI:** 10.1002/jev2.12347

**Published:** 2023-07-21

**Authors:** Giovanni Camussi, Jan Lötvall

**Affiliations:** ^1^ University of Torino Turin Italy; ^2^ Krefting Research Centre, Institute of Medicine University of Gothenburg Gothenburg Sweden

**Keywords:** extracellular vesicles, clinical trails

A recent study by Johnson et al. (2023), published in the Journal of Extracellular Vesicles (JEV), presents the results from a double‐blind placebo‐controlled trial in humans, using platelet‐derived extracellular vesicles (EVs) as a treatment for wound healing. The study has two important merits. Firstly, it tested the clinical safety and potential efficacy of a single subcutaneous injection of EVs in subjects with an experimentally induced skin lesion. Secondly, the study demonstrates that 'Ligand‐based Exosome Affinity Purification' (LEAP) chromatography can successfully isolate platelet EVs of clinical grade from activated platelets. This study provides evidence that clinical trials with EVs are feasible and supports the need for further double‐blind placebo‐controlled trials.

Despite the abundance of preclinical studies showing the regenerative potential of EVs, their therapeutic application in humans has been limited by the lack of a readily scalable method for clinical‐grade EV purification. LEAP‐purified EVs from activated platelets were shown to retain functionalities related to potential regenerative actions with regards to proliferation and migration of dermal fibroblasts and to angiogenesis, both of which are critical components of skin wound healing. Other frequently used methods to isolate EVs include ultracentrifugation and tangential flow filtration, both of which may need the support of additional methods to provide highly concentrated EVs for clinical treatments, making the process both lengthy and cumbersome. The evolution of efficient ways of purifying and concentrating EVs is crucial for their translation into clinical therapeutic use, and perhaps the LEAP technology is an important step forward.

Several clinical trials have been registered by ClinicalTrial.gov using mesenchymal stem cell‐derived EVs to treat patients with different diseases, but only a few of them have reported the clinical results (reviewed in Klyachko et al., 2020; Leyfman et al., 2023). Unfortunately, most clinical trials with MSC EVs fail in design, by not being double‐blind and placebo‐controlled, which is crucial for concluding whether the regimen tested is effective. In the current study, the authors can conclude that the regimen used failed to provide improved wound healing with the protocol used, as it was both blinded and controlled. This study, therefore, provides an important baseline for any future studies investigating the efficacy of EVs in a clinical setting.

The present study (Johnson et al., 2023) suggests that a single subcutaneous administration of allogenic EVs had a good safety profile in healthy volunteers, which is a positive step forward for those developing EVs for clinical utility. However, it is important to confirm that with repeated EV doses, the safety profile is maintained and to determine if that is the case whether the treatment is given locally or systemically. Therefore, to further establish their safety profiles, prospective double‐blind studies of repeated EV administrations, both locally and systemically, in humans is necessary.

The authors also observed no differences in the recovery times between placebo injection and allogeneic‐platelet EVs injection in healthy individuals. From the design of the present study, it is not possible to draw conclusions about wound healing in an individual where efficient tissue regeneration is absent. It is well known that the healing process is conditioned by many systemic and local factors and that in pathological conditions healing is characterized by impaired tissue regeneration. In such conditions the EVs could be beneficial by reactivating a regenerative process. Indeed, in a recent case‐control interventional study, repeated local administration of autologous serum‐derived EVs for the treatment of chronic venous ulcers, unresponsive to conventional treatments, did show a significant improvement in recovery (Gibello et al., 2023). Serum EVs, which are pro‐angiogenic, are enriched in platelet and endothelial derived EVs and the histological analysis of treated chronic venous ulcers showed active angiogenesis with increased vessel density and collagen deposition. In this study, as the EVs were autologous, patient selection was performed based on an in vitro potency test. We can expect that whereas only some patients may benefit from autologous EVs, allogeneic EVs derived from a pool of healthy donors may be significantly more effective with the potential to become a standardized product to treat many patients. In another placebo‐controlled study with topical MSC‐derived EVs, a beneficial effect could be observed on wound healing after facial laser therapy (Kwon et al., 2020). Thus, the overall evidence suggests that EVs from certain sources may have wound‐healing capacity, not only in experimental animals, but also in humans.

The study by Johnson et al. (2023) on allogeneic platelet‐derived EVs supports the concept that clinical placebo‐controlled double‐blind studies with EVs are possible. In addition, it provides evidence that scalable Good Manufacturing Practice (GMP) grade purification of EVs is feasible. Further clinical studies are needed to determine the efficacy and safety of different types of EVs in the treatment of pathological skin ulcers or wounds in humans, and to document the safety of repeated allogeneic EV administration. We are of the view that any such studies should be performed in a double‐blind placebo‐controlled manner.

## CONFLICT OF INTEREST

The authors declare no conflicts of interest.
